# Isolated Cushing’s Syndrome in Early Infancy Due to Left Adrenal Adenoma: An Unusual Aetiology

**DOI:** 10.4274/Jcrpe.727

**Published:** 2012-09-11

**Authors:** Deep Dutta, Rajesh Jain, Indira Maisnam, Prafulla Kumar Mishra, Sujoy Ghosh, Satinath Mukhopadhyay1, Subhankar Chowdhury

**Affiliations:** 1 IPGMER & SSKM Hospital, Department of Endocrinology & Metabolism, Kolkata, India; 2 IPGMER & SSKM Hospital, Department of Paediatric Surgery, Kolkata, India

**Keywords:** Cushing’s syndrome, infancy, adrenal adenoma, ketoconazole

## Abstract

Bilateral macronodular adrenocortical disease as a part of McCune Albright Syndrome (MAS) is the most common cause of endogenous Cushing’s syndrome (CS) in infancy. Adrenocortical tumors causing CS in infancy are extremely rare. We report the case of a girl with CS who presented at age 4 months with obesity and growth retardation. Her 8 am paired cortisol and adrenocorticotropic hormone levels were 49.3 μg/dL and <1 pg/mL, respectively with non-suppressed serum cortisol (41 μg/dL) on high-dose dexamethasone suppression test. Abdominal computed tomography scan demonstrated a 5.3x4.8x3.7 cm homogenous left adrenal mass with distinct borders. Laparotomy following pre-operative stabilization with ketoconazole 200 mg/day, revealed a 7.5x5x4 cm lobulated left adrenal mass with intact capsule and weighing 115 grams. Histopathology showed small round adrenal tumor cells with increased nucleo-cytoplasmic ratio and prominent nucleoli. The cells were separated by fibrous septae without any evidence of vascular or capsular invasion– findings consistent with adrenal adenoma. On the 8^th^ post-operative day, after withholding hydrocortisone supplementation, the 8 am cortisol level was <1 μg/dL, suggestive of biochemical remission of CS. The patient improved clinically with a 7.5 kg weight loss over the next 3.5 months. This is perhaps the youngest ever reported infant with CS due to adrenal adenoma. Lack of clinical and biochemical evidence of hyperandrogenism as well as the benign histology in spite of the large tumor size (>7 cm diameter; 115 g) are some of the unique features of our patient.

**Conflict of interest:**None declared.

## INTRODUCTION

Iatrogenic hypercortisolism is the most common cause of Cushing’s syndrome (CS) in infancy and childhood (1). In infants and children less than 7 years of age, adrenal tumors and predominantly malignant adrenal carcinoma constitute the most common causes of CS (2,3). Endogenous CS is rare in infancy with less than 100 cases reported to date (4). Except for a few cases of adrenocortical carcinoma or unilateral adenoma (5), almost all the cases of adrenocorticotropic hormone (ACTH)-independent CS in infancy have been reported to be due to bilateral macronodular adrenocortical disease encountered in cases of McCune Albright syndrome (MAS) (6,7,8,9,10). 

CS in infancy tends to be more severe. Typical signs associated with CS may also be absent (4). In infants, generalized obesity with growth retardation, frequently accompanied by virilization, is the most common presenting feature (11). Isolated CS without hyperandrogenism due to adrenal tumor is uncommon in children (12).

We report the case of a girl diagnosed with CS due to a huge left adrenal adenoma who had presented with generalized obesity without virilization at age 4 months. Left adrenal adrenalectomy following control of hypercortisolism with ketoconazole and clinical stabilization resulted in rapid weight loss with clinical and biochemical resolution of CS. 

## CASE REPORT

A 4-month-old female infant was brought to our Endocrinology Clinic with complaints of weight gain in the past 3 months, fever in the past 1 month, and respiratory distress in the past 5 days. The infant was born to non-consanguineous parents at term. Birth weight was 2.8 kg. A maternal history of pregnancy-induced hypertension was reported. 

**Figure 1:**
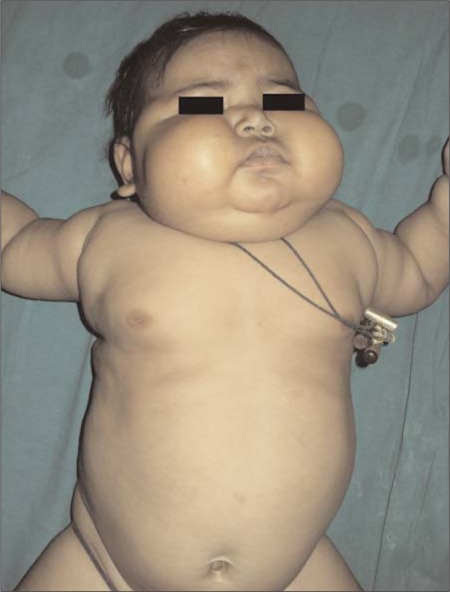
Profile of the patient showing chubby cheeks, moon facies and central obesity

**Figure 2:**
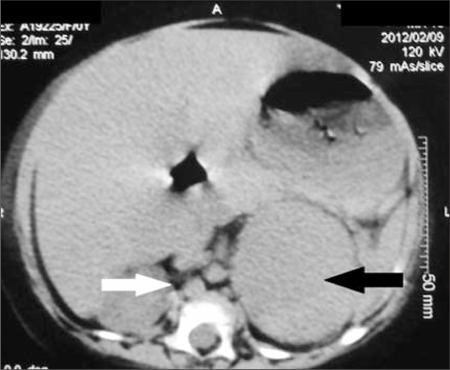
CT of abdomen showing homogenous left adrenal mass with distinct borders (black arrow); contra-lateral adrenal could not be visualized (white arrow)

**Figure 3:**
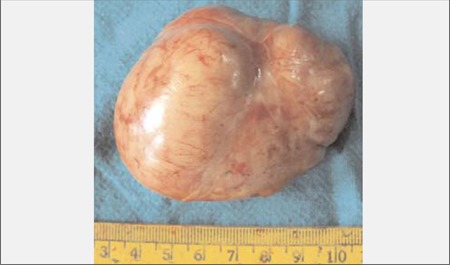
Surgical specimen of resected lobulated left adrenal mass with intact capsule

**Figure 4:**
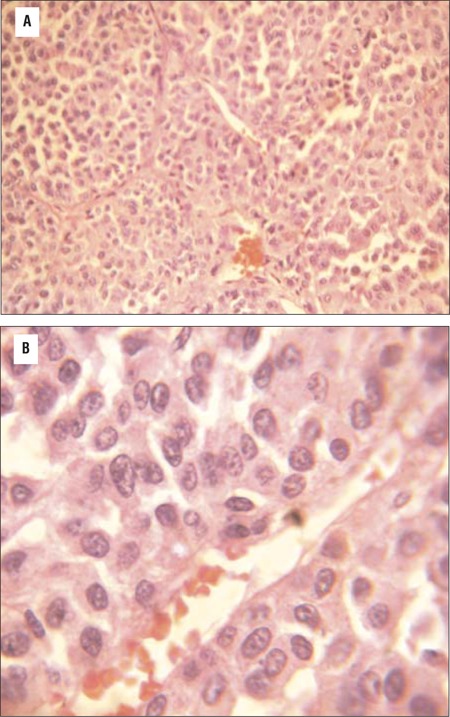
Eosin and hematoxylin staining of adrenal adenoma (40X magnification) showing small round adrenal tumor cells with prominent nuclei and nucleoli, bands of fibrous septae separating the tumor cells, without evidence of vascular invasion; **Figure 4b:** High magnification (100X) showing blue round adrenal cells with increased nucleo-cytoplasmic ratio and prominent nucleoli and no evidence of vascular invasion

Examination revealed a chubby baby with a moon face and a protruding abdomen (Figure 1). Striae, increased body hair, and other stigmata of MAS such as cafe au lait spots were absent. The patient’s body length was 63.9 cm [5-1^0th^ percentile; standard deviation score (SDS):-0.9] and her weight was 15.6 kg (>97^th^ percentile; SDS: +14.6). Her blood pressure was normal. Biochemical evaluation revealed a cortisol level of 49.3 μg/dL with a concomitant plasma ACTH level of <1 pg/mL. Her serum cortisol following high-dose dexamethasone suppression test (HDDST) [0.25 mg of dexamethasone every 6 hours for 48 hours (20 μg/kg/dose)] was 41μg/dL. She had neutrophilic leucocytosis. Blood glucose was mildly elevated (<200 mg/dL), not necessitating insulin infusion (Table 1). The patient was found to have left lower lobe pneumonia. She initially received injections of ceftriaxone combined with amoxicillin-clavulanate for 6 days. Subsequently, she developed diarrhea which improved with oral rehydration. The antibiotic treatment was changed to parenteral piperacillin-tazobactam 1.125 mg thrice a day which she received for 16 days. An abdominal computed tomography (CT) (non-contrast) revealed a 5.3x4.8x3.7 cm homogenous left adrenal mass with distinct borders. The right adrenal was not visualized (Figure 2). Ketoconazole was started at 200 mg/day in 2 divided doses. Laparotomy following resolution of pneumonia and clinical stabilization revealed a 7.5x5x4 cm lobulated left adrenal mass with an intact capsule and weighing 115 g (Figure 3). The inferior vena was spared; there was no lymphadenopathy or adhesions to adjacent organs. Histopathology revealed findings suggestive of adrenal adenoma: small round adrenal tumor cells separated by fibrous septae and with increased nucleo-cytoplasmic ratio, prominent nucleoli, without any evidence of vascular or capsular invasion (Figure 4).

**Figure 5:**
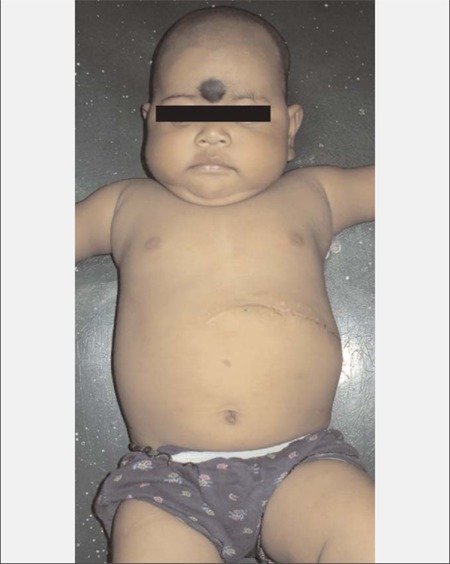
Profile of the patient at 3 months post-operative showing significant weight loss and resolution of features of Cushing’s syndrome

**Figure 6:**
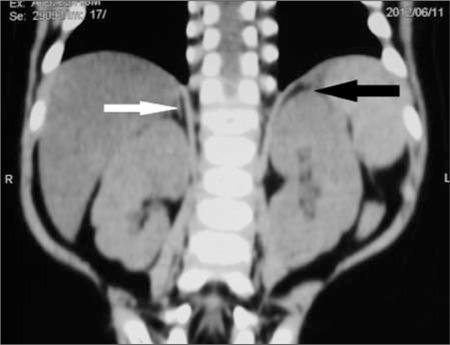
Post-operative (3 months) imaging showing absence of left adrenal (removed) (black arrow) and reappearance of one of the limbs of the right adrenal gland (white arrow)

Post-operatively, the patient received IM hydrocortisone 25 mg 6-hourly for 2 days followed by tapering and shifted to an oral replacement dose of 10 mg in the morning and 5 mg in late afternoon. She also received fludrocortisone 50 μg/day in the first 4 post-operative days. The patient’s blood glucose level normalized by the 4^th^ post-operative day. On the 8^th^ post-operative day, after withholding the late afternoon dose of hydrocortisone the previous day and prior to the administration of the morning hydrocortisone dose, the 8 am cortisol blood level was <1 μg/dL, and was suggestive of biochemical remission of CS. The patient was discharged on post-operative day 14. She had lost significant amount of weight and had reached a weight of 9.6 kg. The hydrocortisone dose was tapered to 5 mg in the morning and 2.5 mg in the late afternoon at 2 months of follow-up when her 1-hour post ACTH (250 μg IM) cortisol level was 6.9 μg/dL (after withholding the previous evening and the same day morning dose of hydrocortisone). Last evaluated three and half months after the surgery, the patient was found to have lost more weight (weight at evaluation: 8.1kg) with resolution of clinical features of CS (Figure 5). Adrenal imaging at this time was normal (Figure 6). Hydrocortisone was stopped with counselling for stress coverage of hydrocortisone as her post ACTH cortisol (using the same protocol) was 10.1 μg/dL.

## DISCUSSION

In this paper, we present an infant with CS who had a large unilateral (left) adrenal mass. Pediatric adrenocortical tumors (ACTs) are rare in infancy and occur primarily in children between one to five years of age (60%), with a peak in incidence below 4 years of age (0.4 cases per million). Nearly half of these ACTs are adrenocortical carcinoma (2). In a registry of 254 pediatric patients with ACTs, 55% presented with virilization alone. Only 5.5% of the children in this registry had isolated CS, and this tended to occur in older children (median age, 12.6 years) (13). Increased androgen production in infancy and early childhood ACTs can be explained by the structure of the adrenal gland at birth. At this time, the inner fetal zone constitutes 85-90% of the gland; the primary steroid product of the inner fetal zone is dehydroepiandrosterone sulphate (14).

Our infant presented with isolated CS without any clinical or biochemical evidence of hyperandrogenism, which is rare. To the best of our knowledge, CS due to adrenal adenoma presenting early in infancy has not been reported previously. Also intriguing is the benign nature of the tumor despite its large size. To control the profound hypercortisolemic state and thus reduce the peri-operative morbidity and mortality, ketoconazole was administered to our patient pre-operatively. Ketoconazole is an imidazole derivative, originally used as an anti-fungal drug but is one of the most commonly used drugs in the medical management of both Cushing’s disease and CS, as well as in preparing these patients for surgery (15). Ketoconazole inhibits several steps of adrenal steroidogenesis including the cholesterol side-chain cleavage enzyme, 17α-hydroxylase and 17,20-lyase, thus has a beneficial effect in CS. In addition, it has a direct inhibitory effect on ACTH release from the pituitary (15,16). In adults, ketoconazole is used in doses of 400-1200 mg/day in divided doses (15). Common side effects are gastrointestinal upset and skin rash. Liver dysfunction in the form of mild reversible increase in transaminase levels can occur in about 10% of the patients (17). However, serious hepatic injury is rare and can rarely be fatal (17,18).

Post-operative hydrocortisone supplementation following surgery for adrenal adenoma causing CS is necessary as the contralateral adrenal gland is usually hypoplastic secondary to prolonged suppressed ACTH secretion from the pituitary due to CS. This explains the lack of visualization of the contralateral adrenal in our patient and the adrenal insufficiency state documented on the 8^th^ post-operative day. 

From the registry of a series consisting of 254 pediatric ACTs, those with completely resected tumors weighing less than 200 g and without metastasis had a five-year event-free survival rate of 91%. Age less than four years was independently associated with better prognosis. A multivariate analysis showed an adjusted odds ratio of 2.6 for patients aged less than four years (13). Complete biochemical resolution of hypercortisolism post-operative with favorable histological features is probably predictive for a good prognosis in our patient. 

To summarize, our patient who presented at age 4 months with obesity and growth retardation is possibly the youngest ever reported case of CS due to adrenal adenoma. Surgical removal of the mass resulted in rapid weight loss with biochemical and clinical resolution of CS.

## Figures and Tables

**Table 1:**
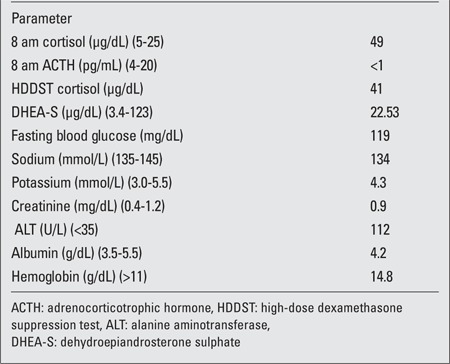
Biochemical findings in the patient
